# Peak exercise SBP and future risk of cardiovascular disease and mortality

**DOI:** 10.1097/HJH.0000000000003008

**Published:** 2021-09-01

**Authors:** Kristofer Hedman, Thomas Lindow, Nicholas Cauwenberghs, Anna Carlén, Viktor Elmberg, Lars Brudin, Magnus Ekström

**Affiliations:** a Department of Clinical Physiology in Linköping, and Department of Health, Medicine and Caring Sciences, Linköping University, Linköping; b Department of Clinical Physiology, Växjö Central Hospital, Clinical Sciences, Clinical Physiology, Lund University, Department of Research and Development, Region Kronoberg, Sweden; c Kolling Institute, Royal North Shore Hospital, University of Sydney, Sydney, New South Wales, Australia; d Research Unit Hypertension and Cardiovascular Epidemiology, Department of Cardiovascular Sciences, University of Leuven, Leuven, Belgium; e Department of Clinical Physiology, Blekinge Hospital, Karlskrona, Lund University, Faculty of Medicine, Department of Clinical Sciences Lund, Respiratory Medicine and Allergology, Lund, Sweden; f Department of Clinical Physiology, Kalmar County Hospital, Kalmar; g Lund University, Faculty of Medicine, Department of Clinical Sciences Lund, Respiratory Medicine and Allergology, Lund, Sweden

**Keywords:** blood pressure, epidemiology, exercise testing

## Abstract

This study aimed to evaluate the risk of all-cause mortality and incident cardiovascular disease associated with peak systolic blood pressure (PeakSBP) at clinical exercise testing.

**Methods:**

Data from 10 096 clinical exercise tests (54% men, age 18—85 years) was cross-linked with outcome data from national registries. PeakSBP was compared with recently published reference percentiles as well as expressed as percentage predicted PeakSBP using reference equations.

Natural cubic spline modelling and Cox regression were used to analyse data stratified by sex and baseline cardiovascular risk profile.

**Results:**

Median [IQR] follow-up times were 7.9 [5.7] years (all-cause mortality) and 5.6 [5.9] years (incident cardiovascular disease), respectively. The adjusted risk of all-cause mortality [hazard ratio, 95% confidence interval (95% CI)] for individuals with PeakSBP below the 10th percentile was 2.00 (1.59–2.52) in men and 2.60 (1.97–3.44) in women, compared with individuals within the 10th--90th percentile. The corresponding risk for incident cardiovascular disease was 1.55 (1.28–1.89, men) and 1.34 (1.05–1.71, women). For males in the upper 90th percentile, compared with individuals within the 10th--90th percentile, the adjusted risks of all-cause death and incident cardiovascular disease were 0.35 (0.22–0.54) and 0.72 (0.57–0.92), respectively, while not statistically significant in women. Spline modelling revealed a continuous increase in risk with PeakSBP values less than 100% of predicted in both sexes, with no increase in risk more than 100% of predicted.

**Conclusion:**

Low, but not high, PeakSBP was associated with an increased risk of mortality and future cardiovascular disease. Using reference standards for PeakSBP could facilitate clinical risk stratification across patients of varying sex, age and exercise capacity.

## INTRODUCTION

Although a drop in SBP during the later stage of exercise is an acknowledged marker for underlying cardiac disease and is associated with a poor prognosis [1–3], the prognostic implication of reaching a high peak SBP remains controversial [4–6]. It is also largely unknown whether the risk associated with a high peak SBP at exercise testing is different between men and women, and whether this risk differs between individuals with either a low or high risk of future cardiovascular disease or with established cardiovascular disease.

Part of the controversy on the risk associated with a high peak SBP may relate to different thresholds to define an exaggerated SBP response [7–10], or the fact that there are several important confounding factors associated with both peak SBP and outcome (such as sex, age, exercise workload and SBP at rest) [6,7,11–13]. Recently, age and sex-specific reference percentiles for peak SBP have been published for treadmill [12] and cycle ergometry [11] exercise testing. In addition, reference equations to calculate predicted peak SBP are available, allowing for evaluation of an individual's peak SBP in relation to sex, age, SBP at rest and exercise capacity [11]. Evaluating the peak SBP response in relation to reference standards rather than absolute values or arbitrary thresholds, may be more adequate in establishing risk for future outcomes, and could shed light upon some of the controversies in previous literature. Due to a lack of reference standards up until recently, this has not yet been investigated.

The primary aim of this study was to determine the risk of future cardiovascular disease and all-cause mortality associated with lower and higher peak SBP than predicted, using recently published sex specific reference standards for peak SBP. Our secondary aim was to explore if the risk associated with having a high or low peak SBP differed between patients with different cardiovascular risk profile at baseline. We hypothesized that both lower and higher peak SBP than predicted would infer a greater risk for all-cause mortality and incident cardiovascular disease.

## MATERIALS AND METHODS

### Study design and participants

**FIGURE 1:**
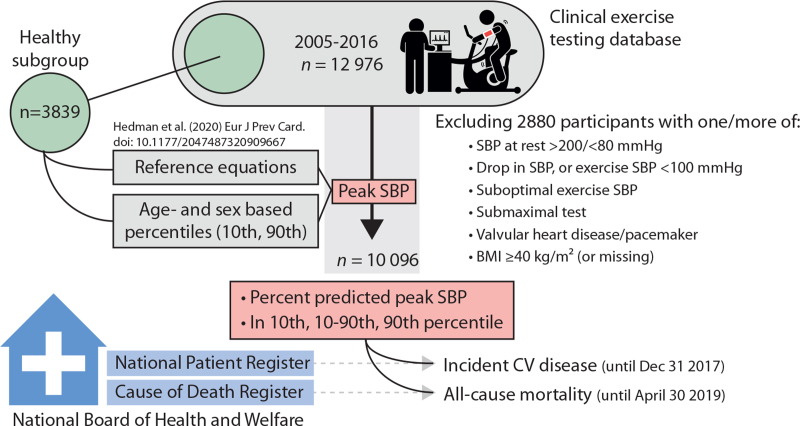
Study design. For each individual, percentage of predicted peak SBP was calculated as well as a categorization of peak SBP according to age and sex-specific 10th and 90th percentiles. For details on exclusion criteria, see text. CV, cardiovascular.

In this retrospective cohort study (Fig. 1), all consecutive individuals aged 18–85 years referred for bicycle exercise testing at the department of Clinical Physiology at Kalmar County Hospital, Sweden, between May 2005 and October 2016 were considered for inclusion (*n* = 12 976).

We excluded individuals with any of the following (criteria not mutually exclusive): a drop in SBP during exercise despite increasing workload (*n* = 296), no SBP measurement during the last 2 min of exercise (*n* = 213), a submaximal exercise test (*n* = 354, defined as a rating < 15 on the Borg scale on rate of perceived exertion), a previous diagnosis of valvular heart disease (*n* = 487), an implanted pacemaker or cardiac defibrillator (*n* = 42), a BMI at least 40 kg/m^2^ or missing BMI data (*n* = 355), a lying or seated resting SBP less than 80 mmHg (*n* = 455) or more than 200 mmHg (*n* = 132), any exercise SBP measure less than 100 mmHg (*n* = 60), only one SBP measurement during exercise (*n* = 321), less than 2 min between first and last SBP measurement during exercise (*n* = 1053), less than 1 min to the first exercise SBP measurement (*n* = 23) or a workload less than 50 Watt at last SBP measurement (*n* = 86).

The study complies with the Declaration of Helsinki and was approved by the Regional Ethical Review board (2012/379–31 and 2018/141–31). Informed consent was waived for this deidentified analysis.

### Exercise test and blood pressure measurement

Details on our exercise testing protocol and equipment have been reported elsewhere [11,14]. In brief, all exercise tests were performed on an electrically braked bicycle ergometer with continuous 12-lead electrocardiographic monitoring. A ramp protocol was used, in most cases starting at 30 Watts (W) in women and at 50 W in men, followed by a continuous ramp of 10 W/min in women and 15 W/min in men. In the absence of any termination criteria (severe chest pain, ST-depression ≥0.4 mV or malignant dysrhythmias), each test was driven with the aim of maximal or near-maximal exertion.

Exercise capacity in percentage was calculated in relation to previously published reference equations [15], accounting for age, sex, height and ramp increment.

Resting SBP and DBP were measured in supine position before exercise, after a few minutes of rest. Seated SBP was measured 1–2 min before exercise commenced. During exercise, SBP was measured every second to third minute in the right arm using a Doppler probe over the radial artery, with manual cuff inflation/deflation, while the individual was instructed to let go of the handlebars with both arms. SBP was recorded at the appearance of the first Korotkoff sound.

Peak SBP was defined as the highest SBP during exercise. Two different approaches were used to relate peak SBP to reference standards (detailed in supplementary Table 1). First, each individual's peak SBP was categorized using the age- and sex-specific lower 10th and upper 90th percentiles, respectively, as ‘low’ (<10th), ‘normal’ (10th--90th) or ‘high’ (>90th). Second, we used sex-specific reference equations that include age, seated SBP at rest before exercise and exercise capacity (in Watt).

### Baseline and comorbidity data

Using the unique Swedish social security number, the exercise test database was cross-linked with the Swedish National Patient Register. This registry contains all in-patient and out-patient hospital diagnoses for each Swedish citizen and its completeness is well established [16]. We retrieved all diagnoses 5 years prior to the exercise test for each individual, coded according to the International Classification of Diseases version 10 (ICD-10), to define prevalent disease and comorbidities. Medications were recorded at the time of the test, as reported by the patient. Prevalent diabetes mellitus, hypertension and hyperlipidaemia were defined as either of a diagnosis per hospital data, or use of any medication relevant for each of the respective diseases. Individuals with an SBP more than 140 mmHg and/or a DBP more than 90 mmHg (lying) prior to the test were also considered hypertensive.

### Outcomes

Survival status was ascertained on 30 April 2019 by cross-linkage with the Swedish Cause of Death Register, maintained at the National Board of Health and Welfare and covering virtually all deaths nationwide. The register is based on death certificate diagnoses, where date and cause of death is reported by a physician or at autopsy.

Incident cardiovascular disease was determined on 31 December 2017 using the Swedish National Patient Register (described above) and was defined as an occurrence of either ischemic heart disease (IHD, ICD-10-codes: I20-I25), heart failure (I50) or cerebrovascular disease (I60-I69) following the exercise test. Incident cardiovascular death was defined as death with an underlying cause of death coded with any ICD-10-code within chapter IX (I00-I99).

### Subgroups and sensitivity analyses

Analyses was performed per sex and per each of three subgroups: ‘Lower risk’: individuals without diagnosed cardiovascular disease and free from diabetes mellitus, hypertension and hyperlipidaemia; ‘With CV risk’: individuals without established cardiovascular disease as per diagnosis, but with any of the risk factors listed above or the use of any cardiac medication; ‘Established CV disease’: individuals with a diagnosis within 5 years prior to the exercise test of HF, IHD, cerebrovascular disease, atrial fibrillation, pulmonary embolism, pulmonary arterial hypertension or cardiomyopathy. For details, see supplementary Table 2. The relative risk of incident cardiovascular disease was analysed for all subjects, as well as in subjects without established cardiovascular disease at baseline only.

Two sensitivity analyses were performed; comparing outcome in individuals with and without hypertension at rest at the time of the exercise test and comparing outcome in subjects with an exercise capacity of at least or below 100% of predicted.

### Patient and public involvement statement

It was not possible to involve patients or the public in the design, conduct, reporting or dissemination plans of our research.

### Statistical analyses

Cross-linking of databases and initial data cleaning were performed using Stata Statistical Software: Release 14.2 (StataCorp. 2015. College Station, Texas, USA: StataCorp LP). Further database management and statistical analyses were performed using R version 4.0.0 (R Foundation for Statistical Computing 2020) and SPSS software, v25.0 (IBM Corp, Armonk, New York, USA). Two-sided statistical significance was set at *P* value less than 0.05 in all analyses. Student's *t*-test was used to compare means and χ^2^ tests were used to compare proportions.

Cox proportional hazards ratios with 95% confidence intervals (95% CI) were calculated for all-cause mortality and for incident cardiovascular disease. Natural cubic spline modelling was used to characterize the risk associated with peak SBP as a continuum, using three knots placed at sex-specific 25th, 50th and 75th percentiles (excluding outlier individuals with a peak SBP in the upper or lower 1st or 99th percentile). For the analysis of incident cardiovascular disease, those with a baseline diagnosis of heart failure, IHD or cerebrovascular disease were excluded.

The models were presented unadjusted as well as adjusted for possible confounding factors, with different adjustments according to analysis as noted in each table and figure legend. Overall, the following variables were considered as confounding factors: age, BMI, SBP at rest, exercise capacity (% of predicted), baseline diagnosis of diabetes mellitus or hyperlipidaemia, baseline cardiovascular disease (heart failure, IHD or cerebrovascular disease), renal disease, chronic obstructive pulmonary disease and the use of beta-blocker medication.

The data underlying this article will be shared on reasonable request to the corresponding author.

## RESULTS

In total, 10 096 individuals (58 ± 14 years, 54% male) were included. Of these, 4793 (47%) had at least one cardiovascular risk factor or comorbidity but no diagnosed cardiovascular disease, while 1196 (12%) individuals had a diagnosed cardiovascular disease prior to the exercise test (Table 1).

**TABLE 1:** Baseline characteristics, per sex and per baseline cardiovascular risk profile

	Per sex		Per CV risk subgroup		
	Male (*n* = 5475)	Female (*n* = 4621)	Free from CV risk factors and disease (*n* = 4107)	With CV risk factors (*n* = 4793)	With established CV disease (*n* = 1196)
Male (%)	5475 (100%)	0 (0%)	2268 (55.2%)	2438 (50.9%)	769 (64.3%)
Age (years)	56 ± 15	60 ± 13	50 ± 14	64 ± 11	65 ± 10
Weight (kg)	86 ± 13	72 ± 13	78 ± 14	81 ± 15	83 ± 15
Height (cm)	179 ± 7	165 ± 6	173 ± 10	171 ± 9	173 ± 10
BMI (kg/m^2^)	27.0 ± 3.8	26.5 ± 4.4	25.8 ± 3.8	27.6 ± 4.2	27.5 ± 3.9
HR_lying_ (1/min)	73 ± 13	75 ± 13	74 ± 13	74 ± 14	71 ± 13
SBP_lying_ (mmHg)	138 ± 18	138 ± 21	124 ± 11	146 ± 19	140 ± 19
DBP_lying_ (mmHg)	80 ± 10	78 ± 10	75 ± 8	81 ± 10	79 ± 10
Diagnosed CV disease before exercise test, *n* (%)					
Heart failure	50 (0.9%)	28 (0.6%)	–	–	78 (6.5%)
Ischaemic heart disease	435 (7.9%)	219 (4.7%)	–	–	654 (54.7%)
Atrial fibrillation/flutter	248 (4.5%)	137 (3.0%)	–	–	385 (32.2%)
Cardiomyopathy	19 (0.3%)	6 (0.1%)	–	–	25 (2.1%)
Cerebrovascular disease	89 (1.6%)	56 (1.2%)	–	–	145 (12.1%)
Risk factors and comorbidities before exercise test, *n* (%)					
Hypertension	2625 (44.9%)	2342 (50.7%)	–	4101 (85.6%)	866 (72.4%)
Diabetes mellitus	323 (5.9%)	179 (3.9%)	–	358 (7.5%)	144 (12.0%)
Hyperlipidaemia	664 (12.1%)	463 (10.0%)	–	693 (14.5%)	434 (36.3%)
COPD	64 (1.3%)	87 (1.9%)	35 (0.9%)	85 (1.8%)	31 (2.6%)
Kidney disease	69 (1.3%)	36 (0.8%)	2 (0.1%)	75 (1.6%)	28 (2.3%)
Malignancy	490 (8.9%)	404 (8.7%)	235 (5.7%)	498 (10.4%)	161 (13.5%)
Self-reported use of medication at exercise test, *n* (%)					
Beta-blocker	1080 (19.7%)	996 (21.6%)	–	1345 (28.1%)	731 (61.1%)
Thrombocyte inhibitor	826 (15.1%)	557 (12.1%)	–	809 (16.9%)	574 (48.0%)
Statins	605 (11.1%)	411 (8.9%)	–	637 (13.3%)	379 (31.7%)
Warfarin/NOAC	136 (2.5%)	56 (1.2%)	–	66 (1.4%)	126 (10.5%)
Any antihypertensive	1321 (24.1%)	1189 (25.7%)	–	1962 (40.9%)	548 (45.8%)
ACEI/ARB	1032 (18.8%)	785 (17.0%)	–	1390 (29.0%)	427 (35.7%)
Ca^2+^-antagonist	390 (7.1%)	325 (7.0%)	–	571 (11.9%)	144 (12.0%)
Diuretic	191 (3.5%)	323 (7.0%)	–	420 (8.8%)	94 (7.9%)
Insulin	26 (0.5%)	17 (0.4%)	–	37 (0.8%)	6 (0.5%)
Other diabetes med.	51 (0.9%)	27 (0.6%)	–	70 (1.5%)	8 (0.7%)

ACEI, angiotensin-converting enzyme inhibitor; ARB, angiotensin receptor blocker; COPD, chronic obstructive pulmonary disease; CV, cardiovascular; HR, heart rate; NOAC, nonwarfarin oral anticoagulant.

Mean exercise capacity of individuals in the lower risk group was 95 ± 17% of predicted, compared with 90 ± 16% in individuals with cardiovascular risk factors (*P* < 0.001) and 84 ± 15% in individuals with established disease (*P* < 0.001, supplementary Table 3).

### Outcome analysis

Overall, 872 (8.6%) individuals died during a median (IQR) follow-up time of 7.9 (5.7) years (80 653 person-years; 10.8 deaths per 1000 person-years). Of these, 200 deaths were due to CV disease. Out of the 9268 individuals with no prior diagnosis of heart failure, IHD or cerebrovascular disease at the time of the exercise test, 1581 (17.1%) were subsequently diagnosed with cardiovascular disease during 5.6 (5.9) years of follow-up (54 838 person-years; 28.8 diagnoses per 1000 person-years). For details, see supplementary Table 4.

### Age- and sex specific reference percentiles

#### All-cause and cardiovascular mortality

**FIGURE 2:**
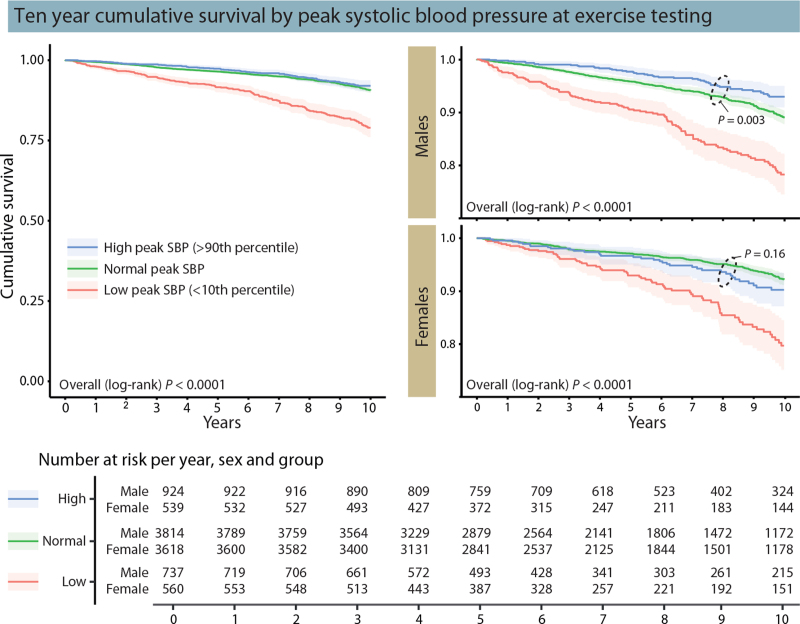
Ten-year cumulative survival by peak systolic blood pressure stratified by age and sex-specific tabulated reference values. Left: survival for all individuals; right: survival per sex; bottom: survival table. Adapted from [11].

In unadjusted analysis, there was an increased risk of all-cause mortality in individuals with an absolute peak SBP in the age and sex-specific lower 10th percentile, but not for individuals in the upper 90th percentile (Fig. 2). In the fully adjusted analysis (Table 2), having a peak SBP in the lower 10th percentile compared to in the 10th to 90th percentile was associated with increased mortality in both men [2.00 (1.59–2.52)] and women [2.60 (1.97–3.44)], while a peak SBP in the upper 90th percentile was associated with a 65% lower risk of death in men [0.35 (0.22–0.54)] but not in women [0.88 (0.63–1.23)]. The risk of cardiovascular mortality associated with low and high peak SBP, respectively, in individuals with and without baseline cardiovascular disease is presented in supplemental Table 5. Overall, the relative risk of cardiovascular mortality was similar to that for all-cause mortality in the adjusted analyses, although the CIs were wider. In men, free from baseline cardiovascular disease, the risk of cardiovascular death associated with a peak SBP in the lower 10th percentile compared to in the 10th--90th percentile was lower than in all individuals (hazard ratio 1.49 vs. 1.96), and not statistically significant. In contrast, the risk associated with being in the lower 10th percentile was greater in women without baseline cardiovascular disease than in all individuals (hazard ratio 2.95 vs. 2.56).

**TABLE 2:** Risk of all-cause death and incident cardiovascular disease by age- and sex specific upper and lower limits of normal for peak systolic blood pressure

	**Model 1**^a^ (Unadjusted)	**Model 2**^b^ (SBP at rest, lying)	**Model 3**^c^ (+exercise capacity)	**Model 4**^d^ (+ cardiac disease, risk factors and medication)
In upper 90^th^ percentile (reference: within 10^th^–90^th^ percentile)				
All-cause mortality				
All subjects	0.85 (0.66–1.10)	**0.50** (0.39–0.65)	**0.57** (0.44–0.74)	**0.58** (0.45–0.76)
Males	**0.49** (0.32–0.77)	**0.50** (0.38–0.65)	**0.33** (0.21–0.52)	**0.35** (0.22–0.54)
Females	1.34 (0.97–1.84)	0.78 (0.56–1.09)	0.87 (0.62–1.21)	0.88 (0.63–1.23)
Incident cardiovascular disease				
All subjects	1.12 (0.95–1.32)	**0.68** (0.57–0.80)	**0.74** (0.62–0.88)	**0.79** (0.67–0.94)
Males	**1.20** (1.02–1.41)	**0.60** (0.47–0.76)	**0.67** (0.53–0.85)	**0.72** (0.57–0.92)
Females	**1.29** (1.02–1.62)	0.79 (0.62–1.01)	0.85 (0.66–1.08)	0.90 (0.70–1.14)
In lower 10^th^ percentile (reference: within 10^th^–90^th^ percentile)				
All-cause mortality				
All subjects	**2.37** (2.02–2.77)	**3.48** (2.95–4.10)	**2.35** (1.98–2.80)	**2.19** (1.84–2.62)
Males	**2.19** (1.79–2.68)	**3.13** (2.54–3.85)	**2.16** (1.72–2.69)	**2.00** (1.59–2.52)
Females	**2.62** (2.04–3.38)	**3.87** (2.97–5.05)	**2.76** (2.10–3.62)	**2.60** (1.97–3.44)
Incident cardiovascular disease				
All subjects	**1.51** (1.31–1.74)	**2.18** (1.89–2.53)	**1.62** (1.39–1.89)	**1.46** (1.25–1.69)
Males	**1.76** (1.48–2.09)	**2.40** (1.99–2.88)	**1.67** (1.38–2.02)	**1.55** (1.28–1.89)
Females	**1.35** (1.07–1.71)	**1.90** (1.50–2.42)	**1.53** (1.20–1.96)	**1.34** (1.05–1.71)

Data presented as HR with 95% confidence interval. In total, 510 out of 5475 males and 362 out of 4621 females died during follow-up. In total, 927 out of 4934 males and 654 out of 4334 females free from heart failure, ischemic heart disease and cerebrovascular disease at baseline were diagnosed with any of these diseases during follow-up.

a Model 1 unadjusted (age and sex are incorporated in the applied reference values).

b Model 2 adjusted for SBP lying at rest before exercise test.

c Model 3 additionally adjusted for percentage of predicted exercise capacity [15].

d Model 4 additionally adjusted for baseline body mass index, diabetes mellitus, hyperlipidaemia, heart failure, ischemic heart disease, cerebrovascular disease, chronic obstructive pulmonary disease, kidney disease, use of beta blocker medication. Reference values from: Hedman *et al.* Eur J Prev Cardiol. 2020;E-pub March 10; doi: 10.1177/2047487320909667. HR, hazard ratio; SBP, systolic blood pressure.

In sensitivity analyses, the risk of all-cause mortality associated with a low peak SBP was similar in individuals with or without hypertension at rest, while the lower risk associated with a high peak SBP in men was only evident in individuals with hypertension (supplemental Table 6). In individuals with an exercise capacity of at least 100% of predicted, a lower than predicted peak SBP was associated with increased risk of all-cause mortality in men, but not in women (supplementary Figure 1).

Using the threshold suggested by the American Heart Association to define an exaggerated SBP response to exercise (≥210 mmHg in men; ≥190 mmHg in women) [2], individuals with an exaggerated SBP response had greater unadjusted survival than individuals without an exaggerated SBP response (supplementary Figure 2). After full adjustment (corresponding to model 4 in Table 2), the risk of all-cause death was 22% lower in individuals with an exaggerated SBP response than in individuals without an exaggerated SBP response (hazard ratio 0.78, 0.67–0.92).

#### Incident cardiovascular disease

As summarized in Table 2, being in the lower 10th percentile was associated with increased risk of incident cardiovascular disease in fully adjusted analysis, in both men [1.55 (1.28–1.89)] and women [1.34 (1.05–1.71)]. There was a 28% lower risk of incident cardiovascular disease in men [0.72 (0.57–0.92)] but not in women [0.90 (0.70–1.14)], associated with being in the upper 90th percentile. The lower risk associated with a high peak SBP in men was only evident in individuals with hypertension, while the increased risk associated with low peak SBP was evident for all individuals (supplemental Table 7).

### Percentage of predicted peak SBP

#### All-cause mortality

**FIGURE 3:**
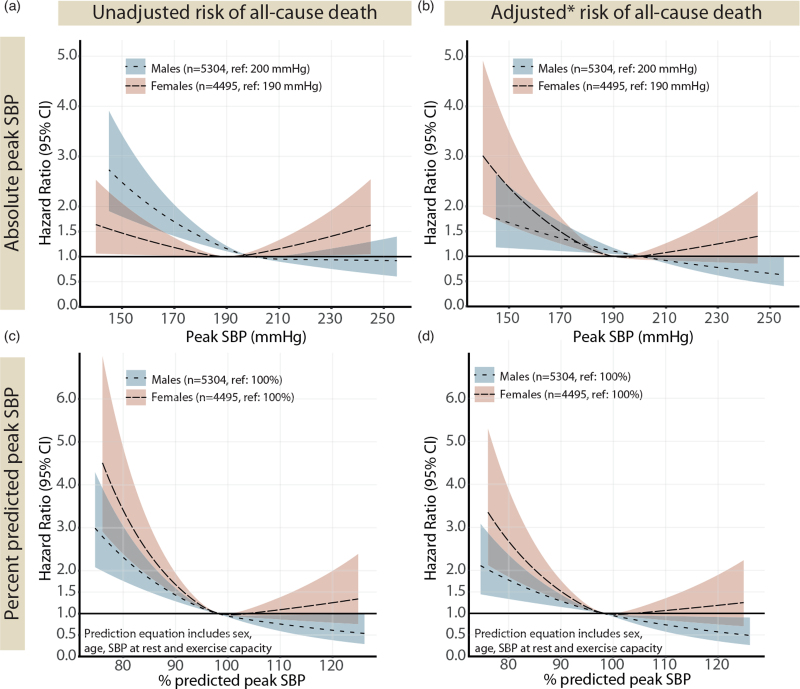
Impact of absolute (a and b) and percentage of predicted (c and d) peak SBP at exercise testing on the risk of all-cause death. The predicted relative risk of all-cause mortality during follow-up, calculated with Cox regression and modelled as natural cubic splines with three knots, excluding individuals in the lower first and upper 99th percentile. (b) adjusted for age, SBP at rest (lying), exercise capacity (% predicted), BMI, diabetes mellitus, hyperlipidaemia, heart failure, ischemic heart disease, cerebrovascular disease, chronic obstructive pulmonary disease, kidney disease, use of beta-blocker medication; (d) adjusted for as in (b) minus age, SBP at rest and exercise capacity (as already included in the reference equation). Percentage of predicted peak SBP are based on sex-specific regression equations in [11]. CV, cardiovascular disease.

The risk of all-cause death associated with peak SBP expressed as percentage of predicted is presented in Fig. 3(c,d). A peak SBP below 100% of predicted was associated with an increased risk of all-cause mortality in both sexes. In individuals free from cardiovascular risk factors and established cardiovascular disease at baseline, a peak SBP below 100% of predicted was similarly associated with an increased risk of all-cause mortality (supplementary Figure 3, a).

**FIGURE 4:**
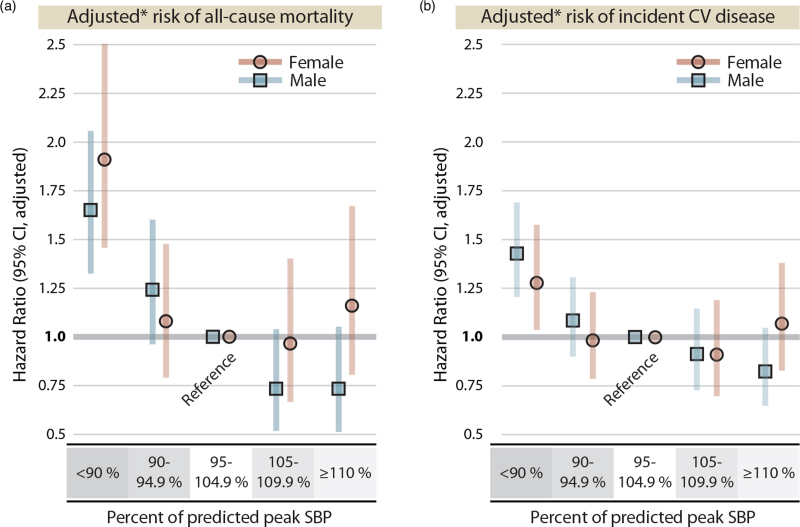
Adjusted risk of all-cause mortality and incident heart failure, ischemic heart disease and cerebrovascular disease based on categories of percentage of predicted peak SBP. (a) adjusted for BMI, diabetes mellitus, hyperlipidaemia, heart failure, ischemic heart disease, cerebrovascular disease, chronic obstructive pulmonary disease, kidney disease, use of beta-blocker medication (age, SBP at rest and exercise capacity are included as covariates in the reference equation). (b) adjusted as in A, minus heart failure, ischemic heart disease and cerebrovascular disease. Percentage of predicted peak SBP are based on sex-specific regression equations in [11].

When grouping individuals into five categories based on percentage of predicted peak SBP (Fig. 4a), the risk of all-cause mortality associated with having a peak SBP below 90% of predicted was 1.91 (1.46–2.50) in women and 1.65 (1.33–2.06) in men, in fully adjusted models.

#### Incident cardiovascular disease

**FIGURE 5:**
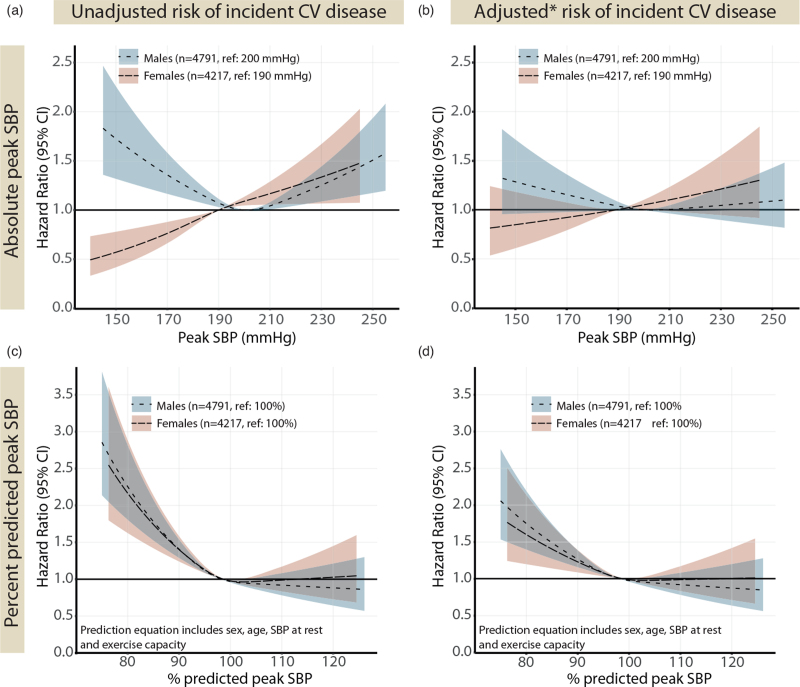
Impact of peak systolic blood pressure at exercise testing on the risk of incident heart failure, ischemic heart disease or cerebrovascular disease. Risk prediction curves modelled as in Fig. 2 but with incident CV disease as outcome and excluding 828 individuals with a baseline diagnosis of heart failure, ischemic heart disease or cerebrovascular disease. Percentage of predicted peak SBP are based on sex-specific regression equations in [11]. (b) adjusted for age, SBP at rest (lying), exercise capacity (% predicted), BMI, diabetes mellitus, hyperlipidaemia, heart failure, ischemic heart disease, cerebrovascular disease, chronic obstructive pulmonary disease, kidney disease, use of beta-blocker medication; (d) adjusted for as in (b) minus age, SBP at rest and exercise capacity (as already included in the reference equation). CV, cardiovascular disease.

The risk of incident cardiovascular disease associated with percentage of predicted peak SBP were similar for men and women, with an increased risk with lower peak SBP, but no increased risk with higher values (Fig. 5). The corresponding models per baseline cardiovascular risk profile are presented in supplementary Figure 3, d--f). The risk of incident cardiovascular disease associated with having a peak SBP below 90% of predicted was 1.43 (1.21–1.69) in men and 1.28 (1.04–1.58) in women, in fully adjusted models (Fig. 4b).

## DISCUSSION

The main finding of this study was that reaching a lower peak SBP than predicted inferred an increased risk of both all-cause mortality and future cardiovascular disease. Importantly, this increase in risk was continuous from values less than 100% of predicted and downward and was evident in both sexes as well as across cardiovascular risk subgroups. Second, there was no increase in risk of all-cause mortality or incident cardiovascular disease in either men or women reaching a high percentage of predicted peak SBP.

Physicians faced with interpreting the SBP response during exercise testing are up for a difficult task, as there is currently no consensus on the normal SBP response to exercise [17], and contemporary reference values have been lacking until recently [11,12,18]. As thoroughly summarized by others [4,5], there is a large variety of criteria to define an exaggerated SBP response to exercise and previous prognostic studies used different, arbitrary cut-offs for peak SBP [7–10]. This, in combination with disparities between the populations studied, has led to results pointing at either a lower [7,8,19,20] or a higher [21–23] risk of cardiovascular events or mortality associated with an exaggerated SBP response. Theoretically, examining the SBP response in relation to well defined reference values rather than using (at the most) sex-specific thresholds would be a more appropriate approach, enabling comparisons across studies and between individuals of, for example, different age. Two recent studies have used reference percentiles to explore the risk of all-cause death associated with peak SBP measured during treadmill exercise in two different U.S. cohorts [18,24]. As discussed, the results are similar to that in the current study, which is the first to study peak SBP from bicycle exercise testing and to use reference equations considering workload.

### A lower than predicted peak SBP

It is well established that exercise-induced hypotension, often defined as either a drop in SBP below the resting value or a drop in SBP after an initial increase [4], is a strong negative prognostic marker of all-cause and cardiovascular mortality [1]. In addition, studies have reported increased risk of events in individuals with only a small increase in SBP with exercise (usually 10–40 mmHg from values at rest) [1,19,20]. Our results extend this, and we show that in reference to predicted peak SBP, there was an increased risk of both all-cause mortality and cardiovascular disease incidence already at a peak SBP corresponding to 90% of predicted. This persisted after additional adjustment for baseline cardiovascular risk factors and comorbidities. Consistently, a peak SBP in the lower 10th percentile as compared to between 10th and 90th percentile, using tabulated reference values considering age and sex, inferred a more than two-fold increase in the risk of all-cause death, and a nearly 1.5-fold increase in incident cardiovascular disease in both sexes after accounting for confounders. Our supplemental analysis revealed similar results for cardiovascular death. The trend of lower peak SBP being associated with higher risk of events persisted for all subgroups based on baseline cardiovascular risk profile, apart from no increase in the risk of future cardiovascular disease in females with lower baseline cardiovascular risk. In contrast, the risk of cardiovascular death associated with a low peak SBP in individuals without established cardiovascular disease at baseline was statistically significant in women (hazard ratio 2.95, 1.38–6.31, but not in men (1.49, 0.78–2.88). Considering the current results, the presence of established cardiovascular disease seems to slightly modify the risk associated with achieving a low peak SBP, and differently so for men and women. However, the overall pattern where a failure to increase SBP to expected levels is coupled to increased risk of mortality and later cardiovascular disease were apparent regardless of baseline CV risk profile.

Our results are similar to those from two recent studies of peak SBP during treadmill testing, using reference standards derived from two different U.S. cohorts. Assaf *et al.* (2021) found a 41% increase in risk of all-cause mortality and a 54% increase in risk of cardiovascular mortality in individuals with a peak SBP in the lowest 10th percentile derived in a healthy reference population (referent: 25th–75th percentile), after adjustment for age, sex and baseline risk factors [18]. Similarly, Hedman *et al.*[24] found an increased risk of all-cause mortality in male U.S. Veterans presenting with a peak SBP falling into the 10th percentile of a different reference population (referent: 10th–90th percentile) after adjusting for age, SBP at rest and exercise capacity. Following further adjustment for baseline cardiovascular risk factors, the increase in risk was no longer statistically significant (hazard ratio 1.07, 0.97–1.18). Taken together, current evidence points to an increased of all-cause mortality, cardiovascular mortality and incident cardiovascular disease associated with having a lower than predicted peak SBP. The current results extend previous results to bicycle exercise testing, and suggest that either of defining a low peak SBP as being in the lower 10th percentile or below 90% of the predicted value, could be used as marker of increased risk of future mortality or cardiovascular disease in both sexes, and regardless of baseline cardiovascular risk profile. Using the percentage of predicted approach, however, enables a continuous grading of the individual patient's risk, and account for exercise capacity within the reference equation.

### A higher than predicted peak SBP

The risk associated with reaching a high peak SBP, as outlined above, has been more controversial. As exercise SBP is physiologically connected to exercise workload, via cardiac output, individuals reaching high workloads are expected to achieve higher peak SBP. Thus, considering workload in interpretation of the SBP response emerge as important [6,7]. In addition, sex, age and SBP at rest have all been found strong predictors of peak SBP [6,11–13] and, importantly, are confounding factors in mortality and cardiovascular outcome analyses. Part of the previous controversy in the literature on whether a high peak SBP is associated with worse prognosis, could lie in the fact that studies linking a high peak SBP to worse outcome were performed in older individuals and/or with a lack of adjustment for age, while studies linking a high peak SBP to future hypertension often were undertaken in a middle-age population [25]. Using a reference equation incorporating sex, age, SBP at rest and exercise capacity, we found no increase in the risk of all-cause mortality or incident cardiovascular disease in either men or women reaching a peak SBP as high as 120% of predicted. Men presented with a continuous *decrease* in risk of all-cause mortality with higher percentage of predicted peak SBP values, as well as a remarkable 65% lower adjusted risk of all-cause death for men with a peak SBP in the upper 90th percentile.

Again, the current results are perfectly aligned with those from the two recent studies from treadmill testing, both reporting a lack of increase in risk of all-cause mortality associated with having a high peak SBP (defined as being in the upper 90th percentile of separate reference populations) [18,24]. In addition, we found a 22% lower risk of adjusted risk of all-cause mortality in individuals having an exaggerated SBP response as defined by the American Heart Association, compared with those without an exaggerated response. It is possible that the reason for the lack of risk increase associated with reaching a higher peak SBP than predicted lies in the physiological coupling to cardiac output and stroke volume [26]. Thus, being able to achieve higher peak SBP would imply adequate cardiac function, when accounting for factors associated with higher peripheral vascular resistance such as SBP at rest, older age and cardiac risk factors.

### Limitations

First, we lack data on individuals’ smoking status, which is a potential confounding factor as it may impact both the SBP response and the risk of future death and cardiovascular disease. However, as smoking is strongly associated with other cardiovascular risk factors, the prevalence of smoking in the lower risk group was probably low. Second, due to the lack of blood sampling data, we relied on previous inpatient or outpatient hospital diagnoses and the self-reported use of medications for defining diabetes and hyperlipidaemia. Third, as the reference values were derived from a highly selected, healthy and geographically homogenous subgroup of the same underlying population as in the current study, our results need validation in other large, ethnically diverse cohorts. Finally, as all tests were performed without analysis of breathing gases, we rely on the subjective rating of exertion in defining tests as maximal or near-maximal.

In conclusion, a peak SBP at clinical exercise testing only slightly lower than the predicted value was associated with increased mortality and risk of incident cardiovascular disease in both men and women. There was no increase in risk associated with higher peak SBP than predicted. Using age and sex-specific reference values in interpreting the SBP response to exercise might facilitate interpretation in exercise testing and improve adverse event prediction in clinical practice.

## ACKNOWLEDGEMENTS

K.H. received general, unrestricted funding from County Council of Östergötland (LIO-822461) and the Swedish Heart and Lung Foundation (20190064). N.C. received a postdoctoral grant from the Research Foundation Flanders (FWO grant 1225021N). M.E. was supported by an unrestricted grant from the Swedish Research Council (2019-02081).

### Conflicts of interest

There are no conflicts of interest.

## Supplementary Material

Supplemental Digital Content
