# Supplementary material for: Peak exercise SBP and future risk of cardiovascular disease and mortality
Source: J Hypertens. 2021 Sep 1;40(2):300–9. doi: 10.1097/HJH.0000000000003008 (PMC8728754; doi:10.1097/HJH.0000000000003008)
Supplement: Supplemental Digital Content [file jhype-40-300-s001.pdf]

SUPPLEMENTARY MATERIAL

For Hedman et al.:

"Peak Exercise Systolic Blood Pressure and Future Risk of Cardiovascular Disease and Mortality"

The following supplementary material has been provided by the authors to give readers additional information about their work:

1. Supplemental Tables

1.1 Reference values used in the analysis 2
1.2 Definition and selection of subjects based on baseline cardiovascular disease profile 3
1.3 Exercise test results per group and sex 4
1.4 Number & proportion of deaths and incident CV disease during follow-up 5
1.5 Risk of CV death based on peak systolic blood pressure 6
1.6 Risk of all-cause mortality per hypertension state at baseline 7
1.7 Risk of incident CV disease per hypertension state at baseline 8

2. Supplementary Figures

2.1 Survival per sex stratified by exercise capacity 9
2.2 Risk of all-cause mortality and CV disease per baseline CV risk 12

SUPPLEMENTARY TABLES

Supplementary table 1. Reference values used in the analysis.		
**Males**		
Predicted peak SBP (mmHg) = 102.3 - (2.33 × Age) + (6.89×10^{-2} × Age^2) - (5.35×10^{-4} × Age^3) + (0.69×$SBP_{sitting}$) + (0.11×$Watt_{max}$)		
**Age group**	**Lower 10th percentile**	**Upper 90th percentile**
18-30	165 mmHg	215 mmHg
31-40	170 mmHg	220 mmHg
41-50	175 mmHg	230 mmHg
51-60	180 mmHg	235 mmHg
61-70	180 mmHg	240 mmHg
71-85	175 mmHg	235 mmHg
**Females**		
Predicted peak SBP = 55.2 + (2.08×10^{-2} × Age^2) - (2.15×10^{-4} × Age^3) + (0.76×$SBP_{sitting}$) + (0.07×$Watt_{max}$)		
**Age group**	**Lower 10th percentile**	**Upper 90th percentile**
18-30	140 mmHg	190 mmHg
31-40	148 mmHg	193 mmHg
41-50	155 mmHg	210 mmHg
51-60	165 mmHg	220 mmHg
61-70	170 mmHg	225 mmHg
71-85	175 mmHg	220 mmHg
Reference equations and percentiles from Hedman K, Lindow T, Elmberg V, Brudin L, Ekstrom M. Eur J Prev Cardiol. 2020;E-pub March 10. Age in years; $SBP_{sitting}$, seated SBP before exercise; $Watt_{max}$, maximal workload (in Watts) achieved. SBP, systolic blood pressure.		

**Supplementary table 2. Definition and selection of subjects based on baseline cardiovascular disease profile.**	
**Group A: Lower risk**	
**Exclusion criteria:**	
Diabetes mellitus	• Medical record diagnosis<br>• Use of insulin or other anti-diabetic drug
Hypertension	• Baseline SBP >140 mmHg or DBP >90 mmHg<br>• Medical record diagnosis<br>• Use of any anti-hypertensive drug (excl. beta-blockers)
Hyperlipidemia	• Medical record diagnosis<br>• Use of statin or other lipid-lowering drug
Cardiovascular disease	• A medical record diagnosis of any of the following:<br>• Heart failure<br>• Ischemic heart disease<br>• Cerebrovascular disease<br>• Cardiomyopathy<br>• Atrial fibrillation/flutter<br>• Pulmonary embolism<br>• Pulmonary arterial hypertension<br>• Use of any cardiac medication
**Group B: With cardiovascular risk factors**	
**Inclusion criteria:**	
Any of the following	• Diabetes mellitus (definition as in group A)<br>• Hypertension (definition as in group A)<br>• Hyperlipidemia (definition as in group A)<br>• Use of any cardiac medication
**Exclusion criteria:**	
Comorbidities	• A medical record diagnosis of any of the following:<br>• Heart failure<br>• Ischemic heart disease<br>• Cerebrovascular disease<br>• Cardiomyopathy<br>• Atrial fibrillation/flutter<br>• Pulmonary embolism<br>• Pulmonary arterial hypertension
**Group C: Established cardiovascular disease**	
**Inclusion criteria:**	
Any of the following	• A medical record diagnosis of any of the following:<br>• Heart failure<br>• Ischemic heart disease<br>• Cerebrovascular disease<br>• Cardiomyopathy<br>• Atrial fibrillation/flutter<br>• Pulmonary embolism<br>• Pulmonary arterial hypertension
Diagnoses retrieved from national hospital database and were retrieved up until five years prior to exercise test. Medication use was self-reported at the time of the exercise test.	

Supplementary table 3. Exercise test results per group and sex.

	Group	Male	Female	P (sex)
**$Watt_{max}$ (Watt)**	Lower risk group	229±51	140±32	<0.001
	With CV risk factors	186±46***	116±27***	<0.001
	Established CV disease	169±40***	106±26***	<0.001
**% pred $Watt_{max}$ (%)**	Lower risk group	94±16	96±18	<0.001
	With CV risk factors	89±16***	92±16***	<0.001
	Established CV disease	83±15***	86±16***	0.008
**HR_{max} (1/min)**	Lower risk group	167±19	160±17	<0.001
	With CV risk factors	147±22***	143±21***	<0.001
	Established CV disease	138±23***	134±23***	0.005
**% Age-pred HR_{max} (%)**	Lower risk group	97±9	96±8	0.008
	With CV risk factors	93±12***	92±12***	0.011
	Established CV disease	89±14***	88±14***	0.30
**Peak SBP_{max} (mmHg)**	Lower risk group	194±21	180±21	<0.001
	With CV risk factors	208±26***	200±24***	<0.001
	Established CV disease	193±27	186±25***	<0.001
**% pred peak SBP (%)**	Lower risk group	98±9	99±9	<0.001
	With CV risk factors	99±10	99±10	0.39
	Established CV disease	95±11***	95±11***	0.47

***, p<0.001; **, p<0.05 for difference vs. lower risk group (one-way ANOVA with Tukey HSD post-hoc test). Groups included 4107 subjects with lower CV risk (2268 males), 4793 with CV risk factors (2438 males) and 1196 with established CV disease (769 males).

Supplementary table 4. Number and proportion of deaths and subjects with incident CV disease during follow-up.					
	**Males** (n=5475)	**Females** (n=4621)	**Lower risk group** (n=4107)	**CV risk factors** (n=4793)	**Established CV disease** (n=1196)
**Death, any cause**	510 (n)<br>9.3 %	362 (n)<br>7.8 %	140 (n)<br>3.4 %	515 (n)<br>10.7 %	217 (n)<br>18.1 %
**Death rate** (per 1000 person-years)	11.8	9.7	4.2	13.5	22.8
**Median (IQR) follow-up time for all-cause death or to 30th April 2019**	7.6 (5.8) yrs	8.1 (5.5) yrs	7.9 (5.7) yrs	7.9 (5.6) yrs	8.1 (6.2) yrs
**Incident IHD, HF or CVD**	927 (n)*<br>18.8 %	654 (n)*<br>15.1 %	282 (n)<br>6.9 %	1201 (n)<br>25.1 %	98 (n)*<br>26.6 %
**Incidence rate** (per 1000 person-years)	32.4*	24.9*	10.7	45.3	5.1*
**Time to incident IHD, HF or CVD or to 31th December 2017**	5.5 (6.0) yrs*	5.8 (5.9) yrs*	6.1 (5.8) yrs	5.3 (5.8) yrs	4.6 (6.0) yrs*

*) for incident IHD, HF or CVD, 828 subjects (541 males) with baseline IHD, HF or CVD were not included in summary statistics for incident disease per sex and for the ‘Established disease’ group (while subjects with baseline arrythmia [n=305], pulmonary embolism [n=54], cardiomyopathy [n=13], arterial thromboembolism [n=4], pulmonary arterial hypertension [n=3] were included).
CV, cardiovascular; IHD, ischemic heart disease; HF, heart failure; CVD, cerebrovascular disease; IQR, interquartile range.

**Supplemental table 5. Risk of cardiovascular death by age- and sex specific upper and lower limits of normal for peak systolic blood pressure.**								
	**Model 1**[a] (Unadjusted)		**Model 2**[b] (SBP at rest, lying)		**Model 3**[c] (+exercise capacity)		**Model 4**[d] (+ cardiac disease, risk factors and medication)	
**In upper 90th percentile (reference: within 10th-90th percentile)**								
Subjects free from baseline heart failure, ischemic heart disease, cerebrovascular disease (n=8900)								
Both sexes	1.27	(0.87-1.88)	**0.65**	(0.43-0.98)	0.77	(0.50-1.17)	0.82	(0.54-1.25)
Males	0.92	(0.49-1.74)	**0.51**	(0.26-0.99)	0.61	(0.31-1.19)	0.68	(0.35-1.36)
Females	1.92	(1.05-3.53)	0.79	(0.40-1.55)	0.90	(0.46-1.78)	0.92	(0.46-1.82)
All subjects (n=10096)								
All subjects	1.04	(0.75-1.45)	**0.58**	(0.41-0.83)	**0.70**	(0.49-1.00)	0.76	(0.53-1.09)
Males	0.80	(0.47-1.38)	**0.47**	(0.27-0.83)	**0.57**	(0.32-1.00)	0.63	(0.36-1.12)
Females	1.61	(0.95-2.73)	0.76	(0.43-1.37)	0.90	(0.50-1.63)	0.97	(0.54-1.76)
**In lower 10th percentile (reference: within 10th-90th percentile)**								
Subjects free from baseline heart failure, ischemic heart disease, cerebrovascular disease (n=8900)								
Both sexes	**2.40**	(1.52-3.78)	**3.36**	(2.12-5.32)	**2.19**	(1.37-3.53)	**1.99**	(1.23-3.23)
Males	**1.96**	(1.08-3.58)	**2.81**	(1.52-5.19)	1.73	(0.92-3.29)	1.49	(0.78-2.88)
Females	**3.56**	(1.70-7.49)	**4.47**	(2.12-9.44)	**3.22**	(1.51-6.86)	**2.95**	(1.38-6.31)
All subjects (n=10096)								
Both sexes	**2.72**	(1.94-3.82)	**3.69**	(2.61-5.19)	**2.50**	(1.75-3.57)	**2.12**	(1.47-3.04)
Males	**2.57**	(1.71-3.85)	**3.61**	(2.38-5.49)	**2.37**	(1.53-3.68)	**1.96**	(1.24-3.08)
Females	**3.46**	(1.86-6.44)	**4.15**	(2.23-7.74)	**2.98**	(1.59-5.61)	**2.56**	(1.34-4.90)

Data presented as HR with 95% confidence interval. In total, 120 out of 5475 males and 80 out of 4621 females suffered from a cardiovascular (CV) death during follow-up. Of the 200 CV deaths, 68 occurred in the group with established CV disease at baseline.
a, Model 1 unadjusted (age and sex are incorporated in the applied reference values);
b, Model 2 adjusted for SBP lying at rest before exercise test;
c, Model 3 additionally adjusted for percent of predicted exercise capacity[1];
d, Model 4 additionally adjusted for baseline body mass index, diabetes mellitus, hyperlipidaemia, chronic obstructive pulmonary disease, kidney disease and use of beta blocker medication. In analysis of all subjects, model 4 was additionally adjusted for baseline heart failure, ischemic heart disease and cerebrovascular disease.
Reference values from: Hedman et al. Eur J Prev Cardiol. 2020;E-pub March 10; doi: 10.1177/2047487320909667. HR, hazard ratio; SBP, systolic blood pressure

Supplemental table 6. Adjusted risk of all-cause mortality in subjects with or without baseline hypertension at rest, by age- and sex specific upper and lower limits of normal for peak systolic blood pressure.

	**No baseline hypertension** (n=5129)		**With baseline hypertension** (n=4967)	
In upper 90th percentile (reference: within 10th-90th percentile)				
All subjects	0.99	(0.46-2.12)	**0.55**	(0.42-0.73)
Males	0.69	(0.22-2.21)	**0.32**	(0.19-0.51)
Females	1.51	(0.54-4.23)	0.85	(0.60-1.21)
In lower 10th percentile (reference: within 10th-90th percentile)				
All subjects	**2.48**	(1.85-3.32)	**2.08**	(1.66-2.61)
Males	**2.23**	(1.54-3.23)	**1.92**	(1.42-2.60)
Females	**2.74**	(1.66-4.50)	**2.57**	(1.82-3.63)

In total, 510 out of 5475 males and 362 out of 4621 females died during follow-up. Analysis with Cox regression (hazard ratio with 95% confidence interval), adjusted for SBP lying at rest before exercise test, percent of predicted exercise capacity, body mass index, diabetes mellitus, hyperlipidaemia, heart failure, ischemic heart disease, cerebrovascular disease, chronic obstructive pulmonary disease, kidney disease, use of beta blocker medication. Age and sex are incorporated in the applied reference values (in Hedman et al. Eur J Prev Cardiol. 2020;E-pub March 10; doi: 10.1177/2047487320909667.

**Supplemental table 7. Adjusted risk of incident CV disease in subjects free from heart failure, ischemic heart disease and cerebrovascular disease at baseline and with or without hypertension at rest, by age- and sex specific upper and lower limits of normal for peak systolic blood pressure.**				
	**No baseline hypertension** (n=5129)		**With baseline hypertension** (n=4967)	
In upper 90th percentile (reference: within 10th-90th percentile)				
All subjects	0.93	(0.59-1.49)	**0.79**	(0.66-0.94)
Males	0.97	(0.54-1.75)	**0.68**	(0.53-0.89)
Females	0.92	(0.43-1.98)	0.92	(0.71-1.19)
In lower 10th percentile (reference: within 10th-90th percentile)				
All subjects	**1.42**	(1.12-1.80)	**1.51**	(1.23-1.85)
Males	**1.44**	(1.07-1.93)	**1.70**	(1.30-2.22)
Females	1.36	(0.91-2.03)	**1.40**	(1.02-1.92)
In total, 927 out of 4934 males and 654 out of 4334 females free from heart failure, ischemic heart disease and cerebrovascular disease at baseline were diagnosed with any of these diseases during follow-up. Analysis with Cox regression (hazard ratio with 95% confidence interval), adjusted for SBP lying at rest before exercise test, percent of predicted exercise capacity, body mass index, diabetes mellitus, hyperlipidaemia, heart failure, ischemic heart disease, cerebrovascular disease, chronic obstructive pulmonary disease, kidney disease, use of beta blocker medication. Age and sex are incorporated in the applied reference values (in Hedman et al. Eur J Prev Cardiol. 2020;E-pub March 10; doi: 10.1177/2047487320909667.				

SUPPLEMENTARY FIGURES

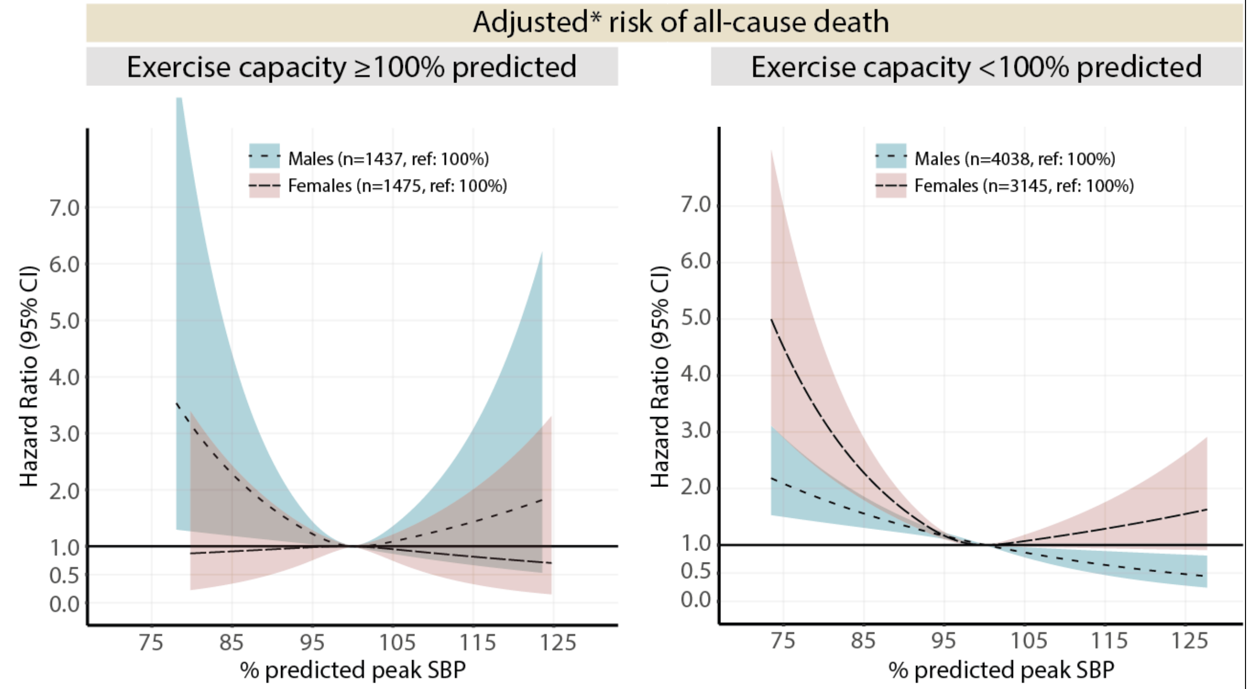

Supplementary figure 1. Survival per sex stratified by exercise capacity of at least (left) or below (right) 100% of predicted. Models adjusted for body mass index, diabetes mellitus, hyperlipidaemia, heart failure, ischemic heart disease, cerebrovascular disease, chronic obstructive pulmonary disease, kidney disease, while age, sex, SBP lying at rest and % exercise capacity are included in the reference equation. SBP, systolic blood pressure.

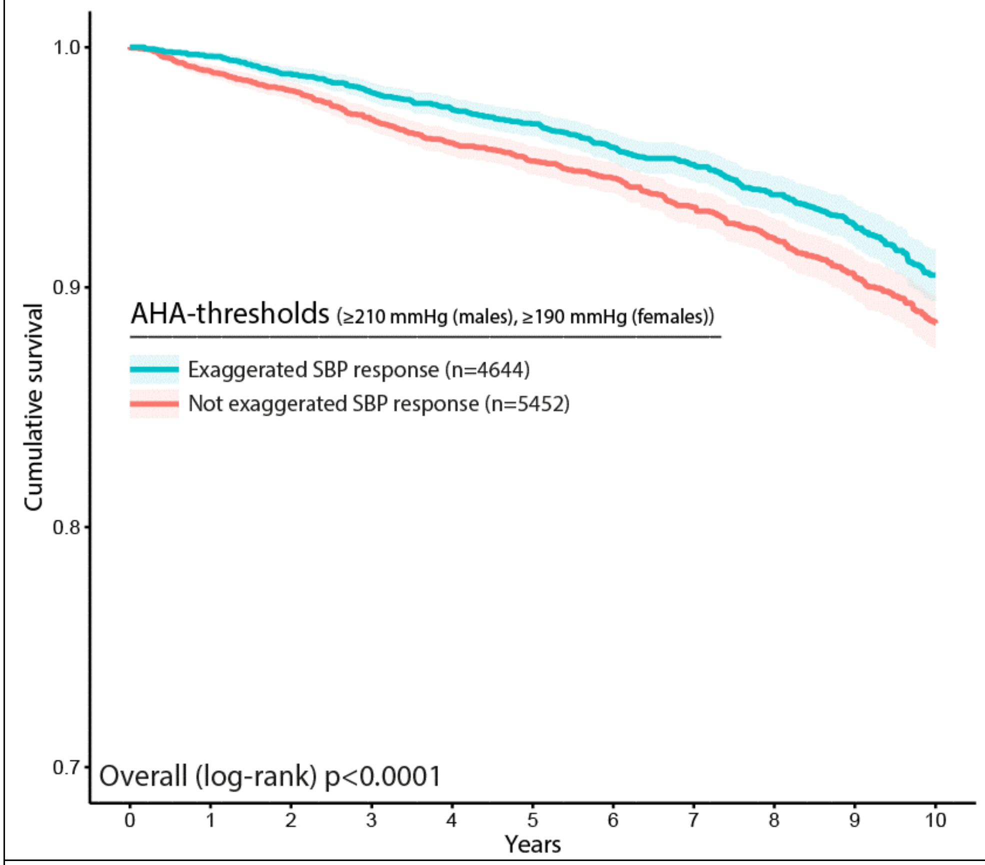

Supplementary Figure 2. Risk of all-cause mortality by AHA thresholds used to define an exaggerated peak SBP response to exercise.
AHA, American Heart Association; SBP, systolic blood pressure.

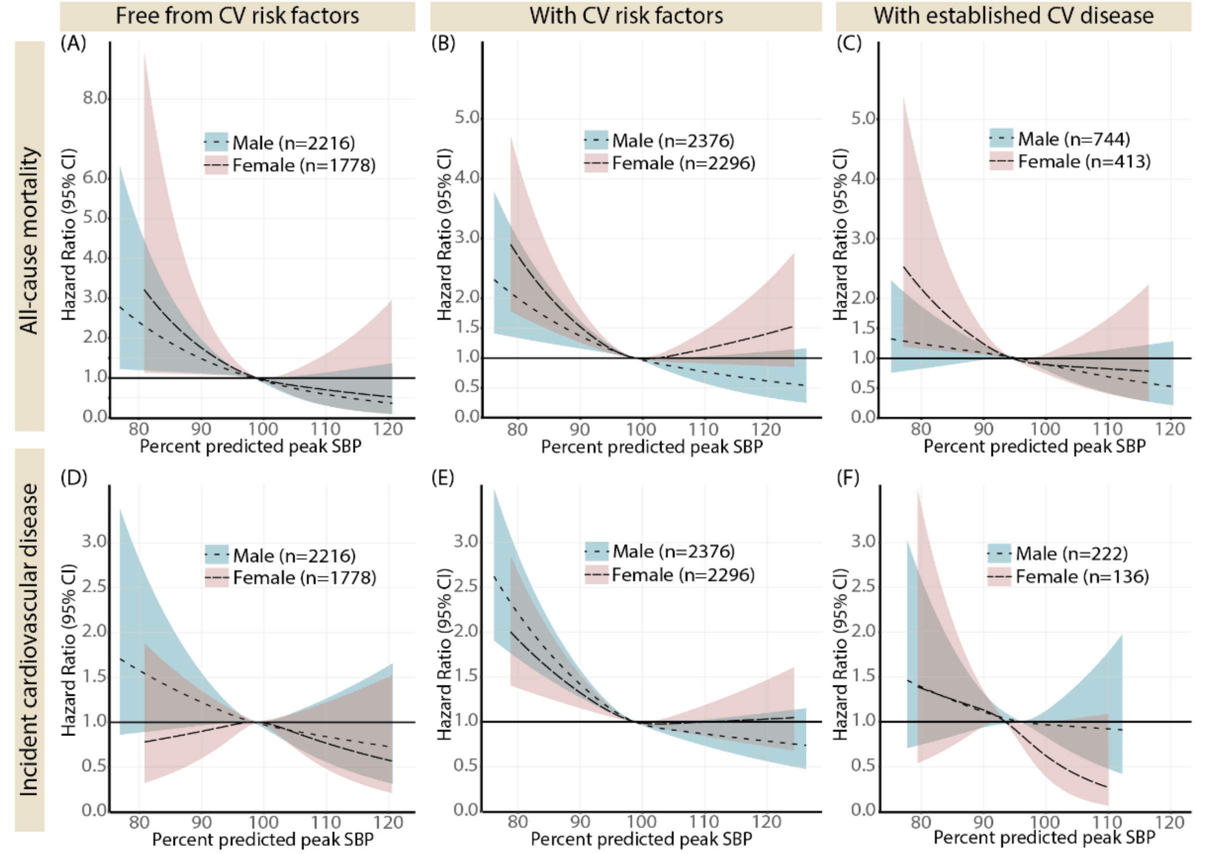

Supplementary Figure 3. Impact of percent predicted peak systolic blood pressure at exercise testing on the risk of all-cause mortality (panel A-C) and on incident heart failure, ischemic heart disease or cerebrovascular disease (panel D-F).
All models are unadjusted albeit the prediction equations include sex, age, systolic blood pressure at rest and exercise capacity in Watt. Survival curves depict Cox proportional hazards modelled as natural cubic splines with three knots at the 25th, 50th and 75th percentile and excluding subjects in the lower 1st and upper 99th percentile. SBP, systolic blood pressure.

1. Brudin L, Jorfeldt L, Pahlm O. Comparison of two commonly used reference materials for exercise bicycle tests with a Swedish clinical database of patients with normal outcome. Clin Physiol Funct Imaging. 2014;34(4):297-307.
